# Characterization and comparative genomic analysis of virulent and temperate *Bacillus megaterium* bacteriophages

**DOI:** 10.7717/peerj.5687

**Published:** 2018-12-10

**Authors:** Abdoallah Sharaf, Miroslav Oborník, Adel Hammad, Sohair El-Afifi, Eman Marei

**Affiliations:** 1 Genetic Department, Faculty of Agriculture, Ain Shams University, Cairo, Egypt; 2 Institute of Parasitology, Biology Centre, Czech Academy of Sciences, České Budějovice, Czech Republic; 3 Faculty of Science, University of South Bohemia, České Budějovice, Czech Republic; 4 Department of Microbiology, Faculty of Agriculture, Minia University, Minia, Egypt; 5 Department of Agricultural Microbiology, Virology Laboratory, Ain Shams University, Cairo, Egypt

**Keywords:** *Bacillus megaterium*, Bacteriophage, Phylogenetic analysis, Comparative genomics

## Abstract

Next-Generation Sequencing (NGS) technologies provide unique possibilities for the comprehensive assessment of the environmental diversity of bacteriophages. Several *Bacillus* bacteriophages have been isolated, but very few *Bacillus megaterium* bacteriophages have been characterized. In this study, we describe the biological characteristics, whole genome sequences, and annotations for two new isolates of the *B. megaterium* bacteriophages (BM5 and BM10), which were isolated from Egyptian soil samples. Growth analyses indicated that the phages BM5 and BM10 have a shorter latent period (25 and 30 min, respectively) and a smaller burst size (103 and 117 PFU, respectively), in comparison to what is typical for *Bacillus* phages. The genome sizes of the phages BM5 and BM10 were 165,031 bp and 165,213 bp, respectively, with modular organization. Bioinformatic analyses of these genomes enabled the assignment of putative functions to 97 and 65 putative ORFs, respectively. Comparative analysis of the BM5 and BM10 genome structures, in conjunction with other *B. megaterium* bacteriophages, revealed relatively high levels of sequence and organizational identity. Both genomic comparisons and phylogenetic analyses support the conclusion that the sequenced phages (BM5 and BM10) belong to different sub-clusters (L5 and L7, respectively), within the L-cluster, and display different lifestyles (lysogenic and lytic, respectively). Moreover, sequenced phages encode proteins associated with *Bacillus* pathogenesis. In addition, BM5 does not contain any tRNA sequences, whereas BM10 genome codes for 17 tRNAs.

## Introduction

*Bacillus megaterium* (Firmicutes) is ubiquitous in nature. It is recognized as an endophyte and a potential biocontrol agent for plant diseases (Kildea et al., 2008). Furthermore, it is known to produce penicillin, amidase, various baking amylases and is used for the biotechnological production of pyruvate, vitamin B12, and so on (Bunk et al., 2009). In alkaline soils, *B. megaterium* plays a significant role in making insoluble phosphorus accessible to plants via the production of CO_2_ and organic acids, which increases soil acidity and the fraction of solubilized phosphates. Certain *B. megaterium* strains even exhibit nitrogen fixation capability (Bergey, 2009). Bacteriophages (phages) are the most abundant biological entities present on the planet (Hambly & Suttle, 2005). Their capacity to infect and kill their hosts makes them an important factor driving the bacterial evolution and preservation of the ecological balance (Chan, Abedon & Loc-Carrillo, 2013; Singh, Poshtiban & Evoy, 2013). Phages can be utilized for various purposes, such as therapy for bacterial infections, for biocontrol of pathogens as protein exposure systems, and for bacterial typing (Haq et al., 2012). A number of phages infecting the genus *Bacillus* have been identified so far (Zeigler, 1999; Lee et al., 2011). *Bacillus*-specific phages demonstrate great diversity with regard to morphology, genome sequence length, gene content, and host range, exhibiting high variability among and within the species of this genus. Moreover, numerous bacteriophages that infect and lyse *B. megaterium* have been studied (Cooney, Jacob & Slepecky, 1975; Carvalho & Vary, 1977; Vary & Halsey, 1980; Van Elsas & Penido, 1982; Serwer et al., 2007; Eppinger et al., 2011). These phages possess double-stranded, linear DNA genomes (Van Elsas, Linhares & Penido, 1982).

Furthermore, the expansion of next generation sequencing (NGS) technologies (Eisenstein, 2012; Klumpp, Fouts & Sozhamannan, 2012), in addition to the possibility of sequencing entire genomes or transcriptomes more efficiently and economically, rather than using the Sanger sequencing strategy, has allowed for obtaining full genomic sequences for a wide range of species. Therefore, the advent of NGS technology provides new opportunities for sequencing of a broad range of organisms, including bacteriophages, quickly, reliably, and considerably inexpensively (Hatfull, 2008).

Here, we shed light on the biological features, genome sequence and annotation of temperate and virulent *B. megaterium* bacteriophages, representing two different groups according to their host range and amplified fragment length polymorphism (AFLP) profile (Elmaghraby et al., 2015). Both phages display different thermal inactivation points (82 and 78 °C) and pH tolerant range (5–9.2 and 5–8.4 pH) while having the same longevity *in vitro* (192 h) (Elmaghraby et al., 2015). Electron microscopy proved that both phages belonged to the *Myoviridiae* family (Elmaghraby et al., 2015). Furthermore, in this paper, we present an updated phylogenetic analysis of *Bacillus* bacteriophages based on the amino acid sequence of terminases.

## Materials and Method

### Bacteriophages

Two isolates of *B. megaterium* phages, namely BM5 and BM10, were obtained from soil. Their biological and morphological properties were reported previously (Elmaghraby et al., 2015). The high titer phage suspension of each isolate was prepared using a liquid culture enrichment technique (Marie, 2013). Fifteen ml of the same high titer phage suspension for each phage was ultra-centrifuged at 30,000 rpm for 90 min at 4 °C in a Beckman L7-35 ultracentrifuge. The pellet was gently re-suspended in 0.5 ml of a 0.2 M potassium phosphate buffer, with a pH level of 7.2 (Lanningan & Williams, 1982). A single-step growth experiment was performed with minor modifications, as reported previously, to determine the latent and rise period and the phage burst size (Pajunen, Kiljunen & Skurnik, 2000; Sillankorva, Neubauer & Azeredo, 2008). Furthermore, 1 ml of the phage suspension (10^10^ pfu/ml) was mixed with 1 ml of exponential phase culture of *B. megaterium* (10^8^ cfu/ml) and incubated at 30 °C for five minutes for phage adsorption. Subsequently, the mixture was centrifuged at 10,000 rpm for 10 min to remove free phage particles. The pellet was re-suspended in 60 ml of a nutrient broth, and the culture was continuously incubated at 30 °C. Phages were sampled at intervals of 5 to 60 min, and their titers were determined. The burst sizes of the phages were estimated by dividing the bacteriophage titers at the plateau phase using the initial phage titers.

### DNA isolation, library preparation, and whole genome sequencing

Genomic DNA was extracted from the bacteriophages, as described by Irshad, Waqas & Saadia (2012), with minor adjustments. The purity and concentration of the DNA was evaluated using a Nanodrop Bioanalyzer N1000 (Thermo Scientific, Waltham, MA, USA). In addition, sequencing libraries were prepared by shearing 1 µg of the phage DNA, to generate blunt-ended fragments, after which the Ion adapters were linked using an Ion Xpress™ Fragment Library Kit (Life Technologies, Carlsbad, CA, USA), in accordance with the manufacturer’s instructions. The produced fragments were amplified employing the Ion OneTouch 200 Template kit (Life Technologies, Carlsbad, CA, USA). Furthermore, libraries were sequenced on an Ion Torrent PGM semiconductor sequencer using the Ion Torrent 314 chip, by applying the standard protocol (Life Technologies, Carlsbad, CA, USA).

### Sequence assembly and annotation

Raw sequencing reads were trimmed and masked using the FASTQ Trimmer and the FASTQ Masker Galaxy Tools Version 1.0.0 (Blankenberg, Gordon & Kuster, 2010), which were assembled by the 454 Newbler Assembler software (Roche Applied Science, IN). The collected contigs were visualized and validated by *Consed* (Gordon & Green, 2013), resulting in the presence of contigs in each phage, which demonstrated a 60-fold sequence read coverage approximately. In addition, sequence homology analysis and assignment to the phage clusters were performed using BLASTn against the *Bacillus* phage database (http://bacillus.phagesdb.org/: 09/2016) (Altschul et al., 1990). The genome sequences of each phage were annotated using the NCBI Prokaryotic Annotation Pipeline. In addition, protein alignment was used for gene predictions to guarantee the consistency of the annotation for closely related genomes. ProSplign (protein aligner software) was utilized to align all the protein sequences. For further refinement, frameshifted alignments and partial alignments were processed using a gene prediction program (GeneMarkS+), and final annotations were established by searching against bacterial and bacteriophage proteins in the SwissProt database (Besemer, Lomsadze & Borodovsky, 2001). The gene predictions were verified using the CPT (Center for Phage Technology) Galaxy (https://cpt.tamu.edu/galaxy-pub/). Furthermore, for genome-wide analyses and other searches, a Phamerator software was employed (Cresawn et al., 2011). Subsequently, conserved domains relationships within genes were determined. Proteins were assorted into “Phamilies” (generally referred to as “Phams”), which are groups of proteins that are largely similar to one another. BLASTp and ClustalW were used to determine the pairwise alignment scores (Cresawn et al., 2011). Phage RNA Polymerase and RNP-recognized promoters were located using the extractupstreamDNA software (https://github.com/ajvilleg/extractUpStreamDNA), after which the MEME program (http://meme-suite.org) was used for motifs elicitation (Bailey & Elkan, 1994; Bailey et al., 2009).

### Genomic similarity and phylogenetic analysis

Proteome similarity and average nucleotide identity (ANI) values of 16 genome sequences of *Bacillus* phages, which were retrieved from GenBank, were determined using JSpecies 1.2.1 (http://imedea.uib-csic.es/jspecies/download.html) at the default BLASTp threshold of score 75 (Richter & Rosselló-Móra, 2009). Gepard was used to create dot plots (Krumsiek, Arnold & Rattei, 2007). To simplify the dot plot analyses, certain phage genomes were reverse complemented, and new sequences were generated to re-orient on the basis of the majority of the phages. A maximum likelihood phylogenetic tree was computed by the RAxML program and by utilizing the lg model (Stamatakis, 2014). The Bayesian tree was calculated by the PhyloBayes program, with both the lg and the C20 models (Lartillot, Lepage & Blanquart, 2009) based on the terminase protein sequence. The supporting values from both methods were merged into a one-rooted tree while the Alloherpesviridae sequences (Anguillid and Cyprinid herpesvirus) were used as the out-group.

### Nucleotide sequence accession numbers

The complete genome sequences of phages BM5 and BM10 were deposited in the NCBI GenBank database under accession numbers KT995479 and KT995480, respectively. Moreover, the raw sequencing data were published in the Sequence Read Archive (SRA) database under the accession number SRP136802.

## Results

### Single-step growth curve

**Figure 1:**
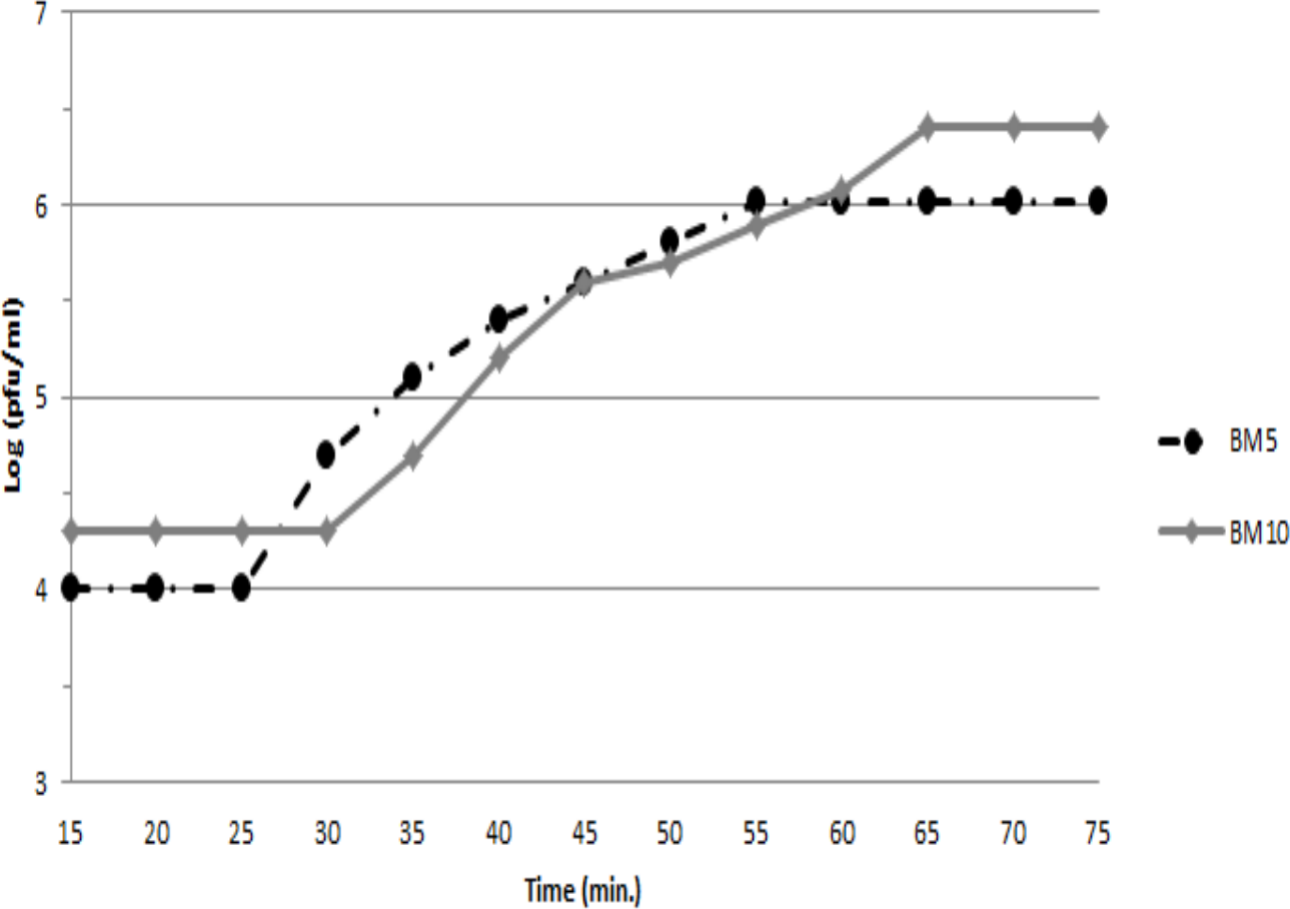
Single-step growth curve of BM5 and BM10 bacteriophages.

To assess the ability of both phages (BM5 and BM10) to lyse *B. megaterium*, the latent and rise periods and the burst sizes of each phage were determined by a single-step growth curve analysis (Fig. 1). The latent periods for BM5 and BM10 phages were estimated to be 25 min and 30 min, respectively. The rise period for both phages was 45 min. The calculated burst size was 103 pfu/cell and 117 pfu/cell, for the BM5 and BM10 phages, respectively.

### Genome organization

**Figure 2:**
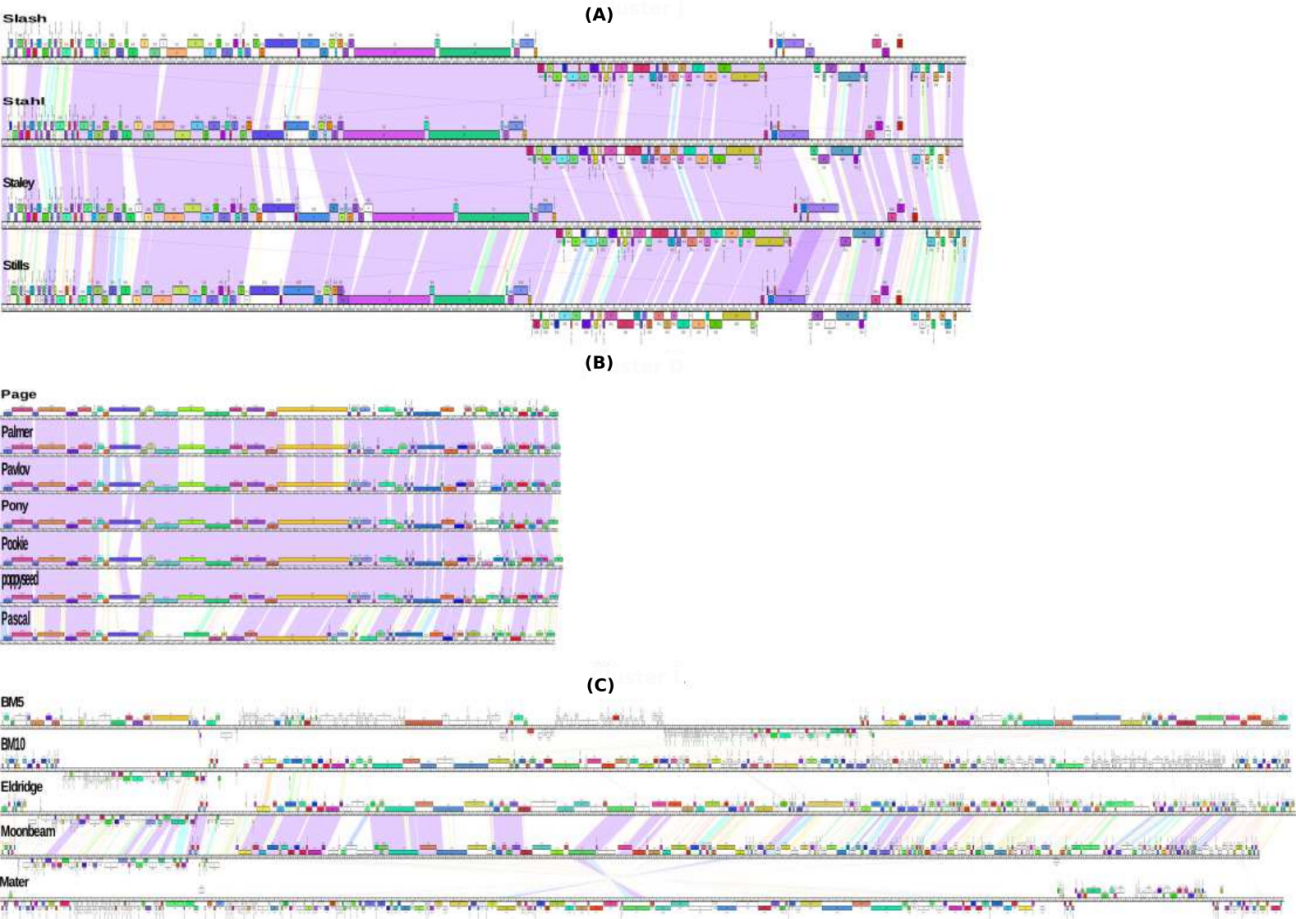
Genomic organization of Bacillus megaterium phages. Phages mapped using Phamerator (Richter & Rosselló-Móra, 2009); the purple lines between phages underline the regions with high similarity, while the ruler corresponds to genome base pairs. The predicted genes are presented as boxes either above or below the genome (line), depending on whether are rightwards or leftwards transcribed, respectively. Gene numbers are displayed within each box. Genes colored according to their function categories “phams”. The unfilled boxes assigned to genes that show low similarity (orphams). *B. megaterium* phages (A) J cluster and (B) D cluster show a high similarity between its members because all members belong to the same sub-clusters (J1 and D1, respectively). Also (C) L cluster phage members belonging to different sub-clusters show a low similarity.

The genome sequencing generated 267,273 reads for BM5 phage and 288,254 reads for BM10 phage, forming an approximately 60-fold coverage. Assemblies yielded 165 kbp and 165.2 kbp genomes for BM5 and BM10, respectively. A comparison of the translated BLAST (tblastx) data (Altschul et al., 1990) from the two genome sequences revealed that they shared a high level of overall similarity; conserved regions reached 91% identity, distributed within the variable regions and with variability ranging from 23% to 88%. Both phage genomes show the common bacteriophages genome organization, with genes of a similar function clustering together (Fig. 2). The last 30 kb of DNA genome sequences of BM5 encoding, for helicases and helicase related proteins, were highly similar to the DNA genome sequences of BM10 (Fig. 2). Annotation of the BM5 genome revealed 225 ORFs (Open Reading Frames), while the BM10 genome encodes revealed 283 ORFs. A total of 97 of the 225 ORFs (43%) in the BM5 genome and 65 of the 283 (23%) in the BM10 genome were assigned functions in comparison with the identified conserved domains (Marchler-Bauer, Derbyshire & Gonzales, 2015) (Tables S1 and S2). In addition, that we identified 17 genes’ coding for tRNAs in the BM10 genome (Table 1).

**Table 1:** Properties of BM10 phage tRNAs.

**ORF no.**	**Strand**	**Begin**	**End**	**tRNA**	**Anti-codon**
ORF 62	+	29,057	29,148	tRNA-Ser	GCT
ORF 63	+	29,156	29,232	tRNA-Cys	GCA
ORF 64	+	29,274	29,351	tRNA-Asn	GTT
ORF 65	+	29,354	29,428	tRNA-Gly	GCC
ORF 66	+	29,434	29,512	tRNA-Thr	TGT
ORF 67	+	29,516	29,591	tRNA-Glu	TTC
ORF 68	+	29,599	29,677	tRNA-Glu	TTC
ORF 69	+	29,769	29,847	tRNA-Thr	CGT
ORF 70	+	29,849	29,938	tRNA-Ser	TGA
ORF 71	+	30,015	30,093	tRNA-Gln	CAA
ORF 73	+	30,168	30,244	tRNA-Gln	CTG
ORF 74	+	30,339	30,413	tRNA-Gln	TTG
ORF 75	+	30,512	30,588	tRNA-Phe	AAA
ORF 76	+	30,677	30,765	tRNA-Leu	TAA
ORF 77	+	30,765	30,841	tRNA-Ile	GAT
ORF 78	+	30,849	30,935	tRNA-Leu	TAG
ORF 79	+	31,029	31,105	tRNA-Met	CAT

### Phage structure and assembly genes

Genes Bm5_9 and Bm5_62 were identified as encoding for the major capsid protein (TIGR04387) (Choi, McPartland & Kaganman, 2008) and the phage capsid protein (pfam05065) (Sutter, Boehringer & Gutmann, 2008), respectively. They were concurrently thought to be involved in the stabilization of the condensed form of the DNA in phage heads. Genes Bm5_61 and Bm5_184 encode for phage head maturation proteases (Peptidase_U35) (pfam04586), which are also involved in phage head development (Duda, Martincic & Hendrix, 1995). Some genes that are essential for the tail development, such as Bm5_66 (TIGR01725), Bm5_68 (cl21657), and Bm5_76 (pfam05709), were also found. Genes involved in head and tail development, such as Bm10_67 encoding for *Caudovirus* prohead protease (cl01521) (Duda, Martincic & Hendrix, 1995), were identified in both BM5 and BM10 genomes (Tables S1 and S2).

### Cell lysis genes

A number of genes involved in lytic activities, such as the degradation of a bacterial cell wall during host infection, was also identified. Bm5_11, Bm5_82, and Bm5_198, coding for Lysophospholipase (EC 3.1.1.5) (cd00229), Peptidoglycan-binding (PGRP) domain (COG3409), and Pectate lyase 3 (cl19188), respectively, are known to hydrolyze lipids, Peptidoglycan and Pectate (Kamen & Woody, 2002; Akoh, Lee & Liaw, 2004). The Bm5_25 and Bm5_77 ORFs were identified as putative endopeptidases and are most probably D-alanyl-D-alanine carboxypeptidase (Peptidase_M) (EC 3.4.16.4) (pfam13539). They belong to the Peptidase M15 family and Prophage tail endopeptidases (pfam06605), required for the infection of the stationary phase bacterial cells (Lee et al., 2001; Stephen et al., 2013). We also identified Bm10_139 encoding Holin (cl2306), and Bm10_51 coding for Acetylmuramoyl-L-alanine amidase (MurNAc-LAA) (cd02696) (EC 3.5.1.28). The Peptidoglycan endolysin, which lyses the bacterial cell wall during the phage release, was also identified (Vollmer et al., 2008) (Tables S1 and S2).

### DNA replication and metabolic genes

We identified several genes encoding endonucleases as well. Bm5_55 encodes HNH_3 endonuclease (pfam13392) and both Bm5_52 and Bm5_113 code for a restriction endonuclease (cd00085) (Makarova, Grishin & Shabalina, 2006). ORFs with putative roles in DNA replication, repair, and modification were identified in the BM10 phage genome. For instance, Bm10_126 encodes a type II DNA-binding protein (DNABII) (cl00257), which serves as the architectural factor in a variety of cellular processes (Prieto, Kahramanoglou & Ali, 2012). Bm10_131 encodes the 5′-3′ polymerase domain of the DNA polymerase (cl02626) (Lee, Kennedy & Yin, 2009) and Bm10_136 codes for a RecA recombinase (COG0468). Other ORFs, potentially involved in transcription and translation, include Bm10_189 (RNA polymerase sigma factor (RpoS)) (TIGR02980) and Bm10_24 (tRNA-splicing ligase (RtcB) (pfam01139) (EC:6.5.1.3)), which catalyzes the splicing of tRNA, and it may also participate in tRNA repair and recovery from stress-induced RNA damage (Tanaka & Shuman, 2011) (Tables S1 and S2).

### Regulatory genes

Several genes that encode proteins that are likely to regulate host functions, are conserved in the phage genomes derived from the *Bacillus* species. The protein encoded by the Bm5_168 gene, containing a bacterial SH3like domain (cl17036), can be found in 28 of the 93 *Bacillus* phages, including phages obtained from cluster D, F, G, and L (Grose et al., 2014). We found Sigma-70 region 2 (pfam04542) encoded by the Bm10_188 gene, which represents the most conserved region of the entire RNA polymerase sigma-G factor protein and is known to interact with the clamp domain of the largest beta polymerase subunit (Jones et al., 2001) (Tables S1 and S2).

### Genes involved in pathogenesis

The Trimeric dUTP diphosphatase (cl00493) and NTP-Ppase (cd11533) proteins that may show the host pathogenesis are encoded by the Bm10_110 and Bm5_108 genes, respectively. These proteins are commonly present in the bacteriophage genomes and have been made to function as G protein-like regulators, which are required for the transfer of staphylococcal virulence factors (Tormo-Más, Mir & Shrestha, 2010; Tormo-Más et al., 2013). Bm5_70 encoding Sialidase (cd15482) plays a vital role in the phage pathogenesis (Telford et al., 2011) (Tables S1 and S2).

Furthermore, 224 and 266 phage-RNA polymerase (RNP) promoters were identified in the BM5 and BM10 phages, respectively, which were all located between the genes (Tables S3 and S4). Motif analysis conducted in this regard reveals 213 conserved motifs (27 nucleotides) for the BM5 phage, while a shorter conserved motif (21 nucleotides) and with the same motif logo was identified in 254 of the BM10 phage-RNA polymerase promoters (Fig. S1). Moreover, additional eight conserved motifs (27 nucleotides) were identified in the BM10 phage with specific DNA recognition sites for the host (*Bacillus*) transcription. So far, none of the downstream ORFs of this host promoter have been functionally assigned (Fig. S1).

### Temperate lifestyle genes

Only the BM5 encodes genes that are suggestive of a temperate lifestyle, such as Integrase_AP2 (pfam14657), were found in a variety of phage integrase proteins, including the ICEBs1 integrase from the *Bacillus subtilis* (Lee et al., 2007)*.* Furthermore, the BM5 encodes a putative site-specific tyrosine recombinase (cd01189) with a conserved C-terminal catalytic domain of other phage integrases, such as the P1 Cre and the lambda Int (Argos et al., 1986) (Tables S1 and S2).

### tRNA genes and codon usage

Phage genomes usually encode a few or no tRNA genes, because they use the host machinery for the synthesis of proteins encoded in the phage genomes. Seventeen tRNA genes were identified in the BM10 genome, while no tRNA genes were identified in the BM5 genome. The 17 BM10 tRNA genes ranged from 77 to 92 nucleotides in size, with one copy each for tRNA-Cys, tRNA-Asn, tRNA-Gly, tRNA-Phe, tRNA-Ile, tRNA-Met, two copies of tRNA-Ser, tRNA-Thr, tRNA-Glu, and tRNA-leu, and three copies of tRNA-Gln (Table 1). All the tRNAs are located in the region between the HTH_XRE (HTH: Helix-turn-Helix) protein-encoding gene and a phage portal protein gene. The presence of tRNAs have been reported in several other phage genomes and are likely to be supporting the phage protein translation efficiency (Grose et al., 2014) and virulence (Bailly-Bechet, Vergassola & Rocha, 2007).

We compared the codon usage pattern of the BM10 tRNAs with that of its host (*B. megaterium*) using the tRNAscan-SE program (Chan & Lowe, 2016; Lowe & Eddy, 1997). We saw that in 10 cases the phage-encoded tRNAs may significantly enhance translation in the phage (Table S5), based on the amino acid usage and/or the codon usage. The products of two genes, Bm10_123 and Bm10_154, appear to encode the nucleotidyltransferase (El-Arabi et al., 2013) and the tRNAHis guanylyltransferase (Hyde et al., 2010; El-Arabi et al., 2013), which may modify the phage or the host tRNAs. The BM10 phage also includes putative tRNAs (tRNA-Thr^CGT^, tRNA-Gln^CTG^ and tRNA-Phe^AAA^), which could be utilized during the infection of an alternative host (Delesalle et al., 2016).

### The genomic relationship between BM5 and BM10

**Figure 3:**
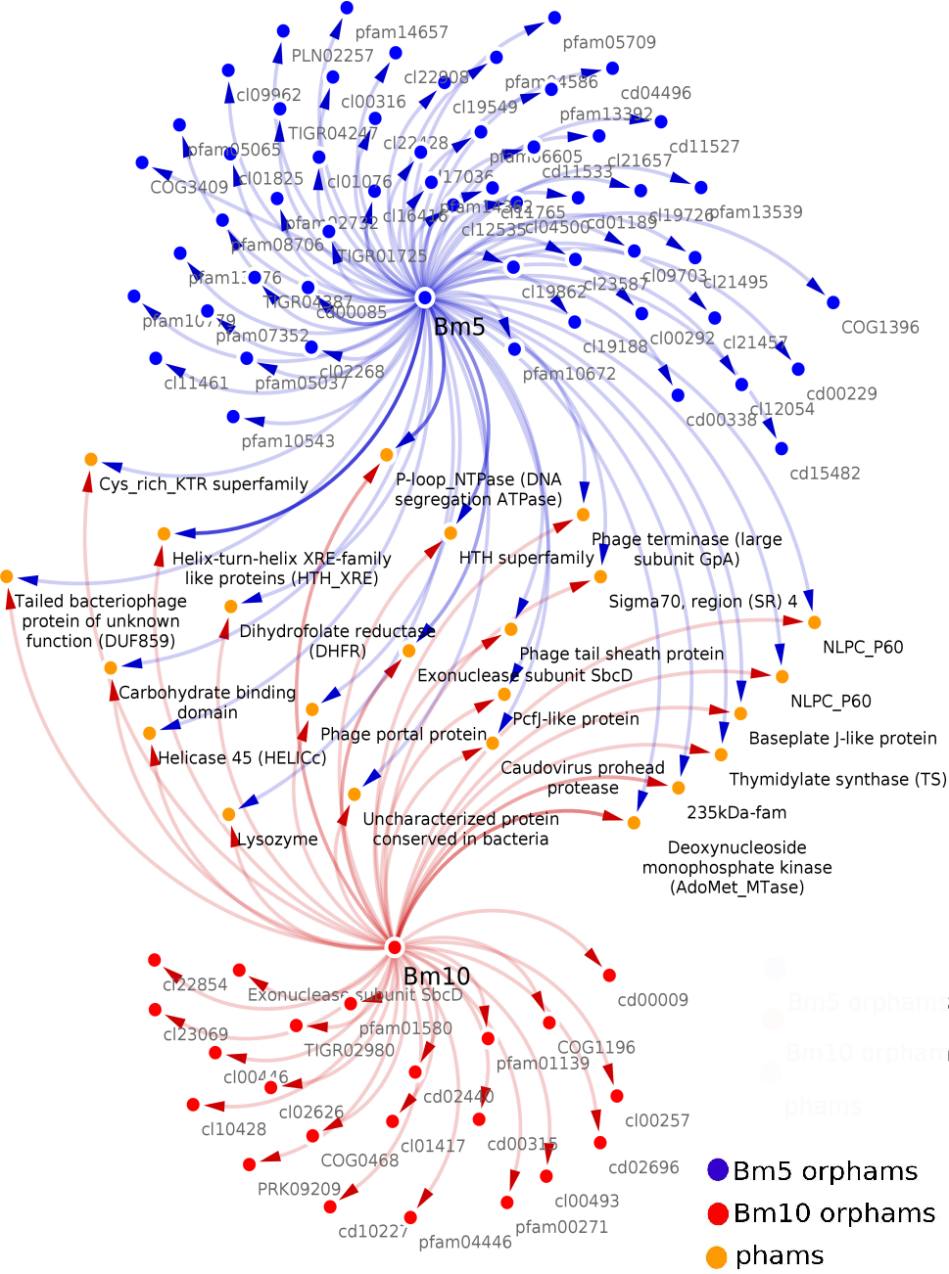
Related Conserved Domains (CD) to the detected Phamilies (phams) using Phamerator. Both BM5 and BM10 orphams (phams containing a gene product from a single phage) presented as blue and red nodes, respectively. While shared phams (orange nodes), located between them. All nodes connected by blue and red arrows represent genes encoding for each pham.

Analysis of the conserved predicted proteins, encoded in the genomes of BM5 and BM10 phages, revealed the huge diversity of these phages, with a total of 407 protein families (phams) of which only 52 (12.8%) were shared by both phages and 355 (87.2%) phams were phage specific or orphams (phams containing a gene product from a single phage). Twenty-one (40.4%) of the 52 shared phams are associated with an identified function (Fig. 3), while the remaining are not.

Bm10_41 and Bm5_159 are members of the pham no.12, assigned to Thymidylate synthase (TS), a well-conserved nucleotide metabolism enzyme identified in 28 *Bacillus* phages (Fig. 3). This enzyme catalyzes the alkylation of the C5 carbon of the pyrimidine nucleotides, known to induce protection against the host restriction system (Sotelo-Mundo et al., 2006). Bm10_121, Bm5_222, and Bm5_223 are members of pham no.58, composed of the Exonuclease subunit SbcD, one of the DNA replicator proteins. Genes encoding structural and assembly proteins were also conserved within the *Bacillus* phages (Fig. 3). These proteins show a high level of sequence conservation across the *Myoviridae*, *Siphoviridae*, *Podoviridae,* and *Tectiviridae* phage families. Identified phams include the phage tail sheath protein, encoded by pham n.35 (Bm10_94 and Bm5_193) and the Baseplate J-like protein, encoded by pham n.51 (Bm10_112 and Bm5_214). These genes have homologs in 33 of the 93 recognized *Bacillus* phages (35%). In addition, Phage terminase, encoded by pham n.18 (Bm10_52 and Bm5_170), and *Caudovirus* prohead protease, encoded by pham n.27 (Bm10_84 and Bm5_184), have homologs in 27 of the 93 investigated *Bacillus* phages (Fig. 3).

Several proteins that regulate host metabolism (including pathogenesis) are also conserved in *Bacillus* phages (Fig. 3). The 235 kDa-fam (TIGR01612) protein, encoded by pham n.43 (Bm10_102, Bm10_104, and Bm5_206), represents a Reticulocyte-binding protein which is localized on the host cell surface and is required in the process of invasion of the parasite (Khan, Jarra & Preiser, 2001). The carbohydrate-binding domain (cl19911), encoded by pham n.52 (Bm10_113 and Bm5_215), is necessary for the carbohydrate-protein interaction (Johnson, Joshi & Tomme, 1996). Two protein phams involved in lysogenic pathways were identified (Fig. 3): Bm10_96 and Bm5_199, members of pham n.37, were identified in three conserved aspartate residues (3D) domain-containing proteins (cd14667), typically found in conjunction with a membrane-bound lytic transglycosylase. Bm10_100 and Bm5_203, members of pham n.41, were assigned to Lysozyme (EC 3.2.1.17) (Fig. 3).

### Comparative genomics of the *Bacillus megaterium* phages

To determine the evolutionary relationship of the 18 sequenced *B. megaterium* phages available in NCBI database, we examined the described phage genomes using complete genome dot plot analysis and pairwise average nucleotide identities (ANI), as previously reported (Hatfull et al., 2010; Pope et al., 2011; Grose et al., 2014). The properties of the 18 sequenced *Bacillus* phages are presented in Table 2.

**Table 2:** Characteristics of reported *Bacillus megaterium* phages with complete genome sequences.

**Cluster**	**Phage name**	**Size (bp)**	**GC%**	**ORFS**	**tRNA**	**Integrase presence**	**Accession number**	**Family**
J1	Slash	80,382	35.23	111	0	Yes	NC_022774.1	*Siphoviridae*
–	Stahl	80,148	35.27	110	0	Yes	NC_028856.1	*Siphoviridae*
J1	Staley	81,656	35.35	113	0	Yes	NC_022767.1	*Siphoviridae*
–	Stills	80,798	35.49	110	0	Yes	NC_028777.1	*Siphoviridae*
D1	Page	39,874	40.71	50	0	No	NC_022764.1	*Podoviridae*
–	Palmer	40,000	40.7	50	0	No	NC_028926.1	*Podoviridae*
–	Pavlov	40,020	40.6	50	0	No	NC_028782.1	–
D1	Pony	39,844	40.7	48	0	No	NC_022770.1	*Podoviridae*
–	Pookie	40,214	40.57	52	0	No	NC_027394.1	*Podoviridae*
D1	poppyseed	39,874	40.71	50	0	No	KF669657.1	–
–	Pascal	39,639	39.9	50	0	No	NC_027372.1	*Podoviridae*
–	BM5	165,030	38.1	224	0	Yes	NC_029069.1	*Myoviridae*
–	BM10	165,213	39.6	283	17	No	KT995480.1	*Myoviridae*
L3	Eldridge	1,650,870	40.4	238	0	No	NC_030920.1	–
–	Mater	164,302	39.5	228	5	No	NC_027366.1	–
–	Moonbeam	161,239	40.2	231	3	No	NC_027374.1	–
–	Silence	40,001	38.3	66	0	No	KT001912.1	–
Singleton	G	497,513	29.93	675	18	Yes	NC_023719.1	*Myoviridae*

**Figure 4:**
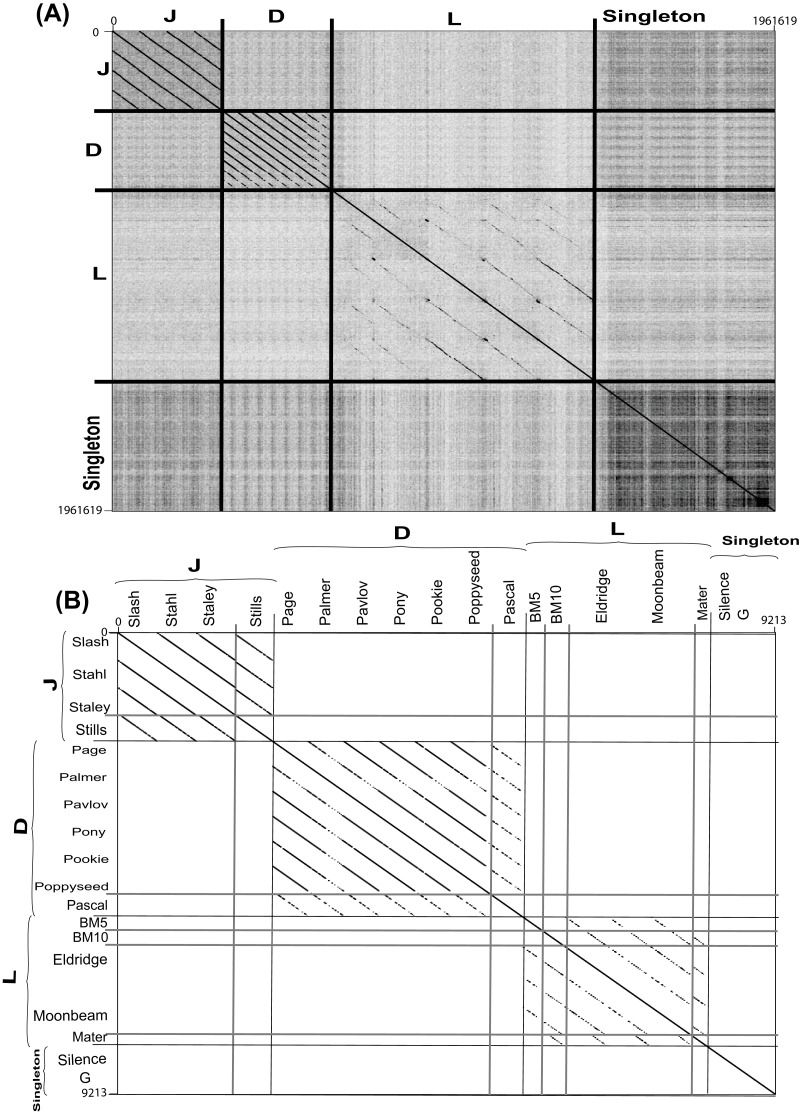
Complete nucleotide genome sequence and single gene amino acid (large terminase) dot plot analysis of *Bacillus megaterium* reveals three clusters (D, L and J) and two singletons. (A) full genome sequence and (B) single gene amino acid. Thick black lines indicate cluster assignments while thick light gray lines indicate cluster assignments, which provided on the *X*- and *Y*-axis. Dot plots created using Gepard (Krumsiek, Arnold & Rattei, 2007). Single gene amino acid sequences were chosen from annotated large terminase gene products.

Each cluster was analyzed by the nucleotide dot plot analysis to reveal groups of high similarity or sub-clusters (Fig. S2). These sub-clusters were selected based on natural divisions in phage similarity seen in the dot plot analysis and defined by ANI similarity values of at least 66% between every two phages within each sub-cluster. Such sub-clustering either represents evolutionary forces that constrain certain types of phages or is an artifact of phage isolation (Grose et al., 2014).

As nucleotide sequences diverge more quickly than amino acid sequences, amino acid dot plot analyses reveal functional conservation. On the other hand, for closely related sequences, the use of nucleotide sequences introduces more bias while building a multiple sequence alignment. Single gene product analysis was performed to determine phage cluster, using a large terminase gene product (TerL), which was identified in all known *Bacillus* phages and has been used for this purpose previously (Grose et al., 2014). A dot plot alignment of the Terminase gene product (TerL) validated our basic cluster/sub-cluster assignment, with all phages grouping in agreement with their clusters or sub-clusters, and the G and Silence phages remaining singletons (Fig. 4B).

### Phylogenetic analysis

**Figure 5:**
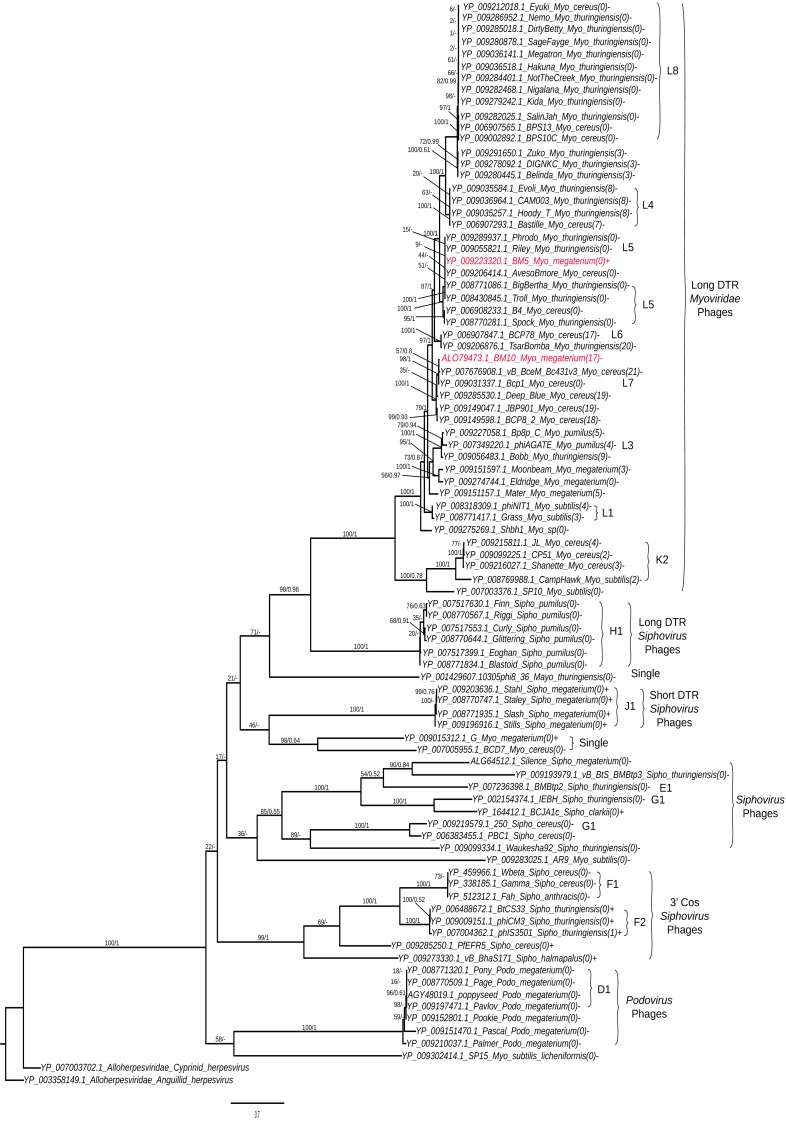
Phylogenetic tree analysis of the *Bacillus* terminase reflects complete genome cluster. Sequenced phages, colored in red. Whole genome sub-cluster assignment and phage families, indicated on the right. Replication approaches for phages also, indicated as a direct terminal repeats (DTR) and cohesive ends (cos). Moreover, the presence and absence of integrase genes as (+) or (−), respectively and number of tRNA genes for each phage were indicated as the number between brackets. The phylogenetic tree were constructed using a Maximum Likelihood distance matrix (Stamatakis, 2014) and Bayesian Monte Carlo Markov Chain (MCMC) matrix (Lartillot, Lepage & Blanquart, 2009). The bootstraps from both methods merged in one rooted tree using the out-group Alloherpesviridae sequences (Anguillid and Cyprinid herpesvirus). Bootstrap values indicate the number of times a node was supported in 2000 re-sampling replications.

Analysis of terminase (TerL) diversity in BM5, BM10 and the other 87 *Bacillus* phages (Fig. 5) provides a robust assignment of their phylogeny (Casjens, 2011) and can be used to interpret the phage replication strategy, (Grose et al., 2014) and to investigate the hybrid generations between phage genera (Canchaya, Fournous & Chibani-Chennoufi, 2003) or families (Recktenwald & Schmidt, 2002). The 89 *Bacillus* phages were grouped into five main clades (Fig. 5).

On the phylogenetic tree, we have indicated the numbers of tRNA genes encoded in each phage order to describe its distribution among the *Bacillus* phages. An association between sub-cluster and number of tRNA genes was been identified. Phages without tRNAs were observed in all sub-clusters—L8, L5, H1, J1, F1, K2, and D1. Phages encoding similar numbers of tRNAs were identified in sub-clusters L1 and L4; the same pattern was observed in *Mycobacteriophages* (Delesalle et al., 2016). An exception was found in the F2 sub-cluster, where only one member (phiIS3501) contained one tRNA gene. It was described as an unusual pro-phage, different from the rest of its family, containing the tRNA-Met gene located upstream of the lysogenic integrase gene (Moumen, Nguen-The & Sorokin, 2012). Also, for identification of the lytic and temperate clusters within the *Bacillus* phage, the presence and absence of integrase genes in each phage was exhibited in the tree. We identified seven lytic sub-clusters (L8, L4, L5, K2, H1, f1 and D1), with all their members lacking the integrase gene, and two temperate sub-clusters (J1 and F2). Interestingly, our sequenced BM5 phage was the only phage containing the lysogenic integrase gene within the L cluster and even within all long DTR *Myoviridae Bacillus* phages. Other temperate phages were previously observed within the lytic *Mycobacteriophages* cluster (Delesalle et al., 2016). To confirm clustering of the BM5 and BM10 phages, a whole genome pairwise ANI analysis was performed using JSpecies 1.2.1 between the BM5 and BM10 phages and other phages clustered in the same clade (Table S6). The ANI percentages between the BM5 phages and the L5 cluster phages (Phrodo, Riley, AvesoBmore, BigBertha, and Troll) ranged from 94% to 98%, while it ranged from 86% to 93% between the BM10 phage and the L7 cluster phages (vB_BceM_Bc431v3 and Bcp1).

## Discussion

Our isolated phages (BM5 and BM10) show low latent period, which is lower than expected for *B. megaterium* (45–60 min) and other *Bacillus* phages (34–145 min) (Brodetsky & Romig, 1965; Colasito & Rogoff, 1969a; Colasito & Rogoff, 1969b; Cooney, Jacob & Slepecky, 1975; El-Arabi et al., 2013; Krasowska, Biegalska & Augustyniak, 2015). This is probably because of the strong activity of endolysins in the BM5 and BM10 phages, which is likely due to mutations in their genes encoding of holin (Stephen et al., 2013) and large burst size, which, in turn, might be caused due to different factors, such as host cell size, culture age, and/or the host physiological conditions (Abedon, Hyman & Thomas, 2003; Choi, Kuatsjah & Wu, 2010; Bolger-Munro, Cheung & Fang, 2013). Moreover, the larger burst size and shorter latency period for both phages reflect their active propagation in the host (Delesalle et al., 2016; Wilson, 1973).

Bioinformatic analyses of the BM5 and BM10 genomes reveal that the genome organization and annotation are in general agreement with other studies of bacteriophages (Grande et al., 2014; Leon-Velarde et al., 2014). A similar mosaic genome structure was observed in most other phage genomes, indicating the extensive horizontal genetic exchange within the phage communities (Rohwer & Edwards, 2002; Van Dessel et al., 2005). Moreover, bacteriophage proteins represent the majority of the members of the identified protein families. The obtained identified genes were involved in phage structuring, DNA replication, nucleotide metabolism, lysis, and the repairing of proteins. The presence of the 17th tRNAs in the BM10 phage genome, which has a larger burst size (117 pfu/cell) than the BM5 phage (103 pfu/cell), along with the absence of tRNAs support the hypothesis that bacteriophages with tRNA genes have a larger burst size as a result of their fast propagation in their hosts (Delesalle et al., 2016; Wilson, 1973). This high number of tRNAs within phage genomes is associated with phage virulence and higher codon usage bias (Bailly-Bechet, Vergassola & Rocha, 2007).

The analysis of the genome sequences of the BM5 and BM10 phages revealed high diversity (66.5% ANI) in these phages. Some of the identified phams can be used in evolutionary and phylogenetic studies. As reported for *Bacillus* phages (Grose et al., 2014), single, ubiquitous, and semi-conserved genes can be utilized for the cluster prediction, particularly, when the whole genome sequence is unavailable. A close evolutionary relationship between the two isolated phages was emphasized by using the 21 identified phams, and it provided information that might prove useful for the comparing the evolution of the gene complement in *B. megaterium* phages and the phylogenetically related *Bacillus* bacteriophages.

Whole genome comparisons of the *B. megaterium* phages generated three clusters (D, L, and J) with genome similarity values of over 50% (Fig. 4A). Two singletons (G and Silence phage) were identified. The ANI values were also estimated within each cluster and determined to have at least 63% similarity of phages within a single cluster. In addition to showing strong evolutionary relationships, whole genome nucleotide dot plots also showed smaller regions of homology (<50% span length) between phages of different clusters. These local homology regions are supposed to be sites of recombination (Fig. 4A). Likewise, the analysis of the proteins conserved between each cluster phage revealed that cluster J, which contained only temperate phages, contained the highest number of conserved proteins (78 phams), whereas 41 and 13 phams were found in the cluster L and D phages, respectively. Interestingly, 20 phams were identified between the BM5 phage and the cluster D phages (Fig. 2). These results are comparable with those described for the 98 *Bacillus* phages by using the same gene product (TerL) (Grose et al., 2014).

The TerL-based phylogenetic analysis supported the clustering and sub-clustering of the genome comparison analysis, and it can be used for conducting further studies of the evolutionary relationships between phage families and phage-packaging strategies. Most *Bacillus* phages that belong to the *Myoviridae* family (specifically, those that belong to clusters H1, K2, L1, L3, L4, L5, L6, L7, and L8) pack their DNA in a similar concatemer structure, thereby resulting in DNAs with long and direct terminal repeats (DTRs). Clusters F1 and F2 *(Siphoviridae Bacillus* phages) are packed with 3′ cos ends. Cluster J of *Bacillus* phages, which belongs to the *Siphoviridae* family, has short DTRs. This type of prediction can be used as a guide to facilitate the experimental determination of tailed-phage chromosome’s end structure. Based on our phylogenetic analysis, we rejected the hypothesis that the lytic phage clusters are more likely to contain tRNA genes than temperate phage clusters that are similar to *Mycobacteriophages* (Bailly-Bechet, Vergassola & Rocha, 2007).

## Conclusion

The present study reports the biological and genome properties of virulent and temperate *B. megaterium* phages. Both the BM5 and BM10 phages displayed high lytic activity to *B. megaterium*. Moreover, both phages lacked repressor determinants to maintain lysogeny by down-regulating lytic promoters and confer superinfection immunity. This increases the potential risk of using *B. megaterium* as a biocontrol and a biofertilizer agent. Moreover, putative tRNAs were identified, thereby revealing the ability of the BM10 phage to infect the other hosts. The first comparative whole genome nucleotide sequences analysis and a large terminase (TerL) protein phylogeny, which reveal the clustering and sub-clustering of *B. megaterium* phages, are presented.

Finally, the study attempts to present the most comprehensive phylogenetic analysis of the *Bacillus* phages, based on terminase amino acids sequences, thereby revealing a robust relationship between the phage families and packing strategies and supporting the fact that the distribution of tRNA genes in the *Bacillus* phage is specific to sub-clusters. On the other hand, our screening of the 87 *Bacillus* phages genomes for integrases and tRNA genes revealed that 66.7% of the 66 lytic *Bacillus* phages lacked tRNA genes while 25% of the 20 temperate *Bacillus* phages contained tRNA genes (Tables S3 and S4). Hence, we do not agree with the assumption that lytic bacteriophages are more likely to contain tRNA genes than temperate bacteriophages.

##  Supplemental Information

10.7717/peerj.5687/supp-1 Table S1 Raw data of BM5 phage, ORFs, summary of homology searches and annotations BM5 phage, ORFs, summary of homology searches and annotations.

10.7717/peerj.5687/supp-2 Table S2 Raw data of BM10 phage, ORFs, summary of homology searches and annotations BM10 phage, ORFs, summary of homology searches and annotations.

10.7717/peerj.5687/supp-3 Table S3 Raw data of identified phage-RNA polymerase (RNP) promoters, location and sequence in BM5 phage genome Identified phage-RNA polymerase (RNP) promoters, location and sequence in BM5 phage genome.

10.7717/peerj.5687/supp-4 Table S4 Raw data of identified phage-RNA polymerase (RNP) promoters, location and sequence in BM10 phage genome Identified phage-RNA polymerase (RNP) promoters, location and sequence in BM10 phage genome.

10.7717/peerj.5687/supp-5 Table S5 Raw data of idetified tRNAs numbers, types and integrase presence in *Bacillus* phage genomes Iidetified tRNAs numbers, types and integrase presence in *Bacillus* phage genomes.

10.7717/peerj.5687/supp-6 Table S6 Raw data of the ANI percentages between BM5 phages, L5 cluster phages and between BM10 phage, L7 cluster phages The ANI percentages between BM5 phages, L5 cluster phages and between BM10 phage, L7 cluster phages.

10.7717/peerj.5687/supp-7 Figure S1 Motif logo for the identified promoters motif logo. (a) BM5 phage-RNP promoters motif logo, (b) BM10 phage-RNP promoters and Bacillus (host)-RNP promoters motif logo Motif logo for the identified promoters motif logo. (a) BM5 phage-RNP promoters motif logo, (b) BM10 phage-RNP promoters and Bacillus (host)-RNP promoters motif logo.

10.7717/peerj.5687/supp-8 Figure S2 Analysis of fully sequenced *Bacillus megaterium* phage genomes belongs to clusters D, J and L Analysis of fully sequenced *Bacillus megaterium* phage genomes belongs to clusters D, J and L.

10.7717/peerj.5687/supp-9 Supplemental Information 1 Raw data for Figure 1 Single-step experiment raw data.

10.7717/peerj.5687/supp-10 Supplemental Information 2 Plaques picture Original Plaques picture of the sequenced phages.

## Additional Information and Declarations

### Data Availability

The following information was supplied regarding data availability: The complete genome sequences of the phages BM5 and BM10 are available at NCBI GenBank: KT995479 and KT995480. The raw sequencing data are available at Sequence Read Archive (SRA): SRP136802.
