# Supplementary figures and images for: Characterization and comparative genomic analysis of virulent and temperate Bacillus megaterium bacteriophages
Source: PeerJ. 2018 Dec 10;6:e5687. doi: 10.7717/peerj.5687 (PMC6292376; doi:10.7717/peerj.5687)

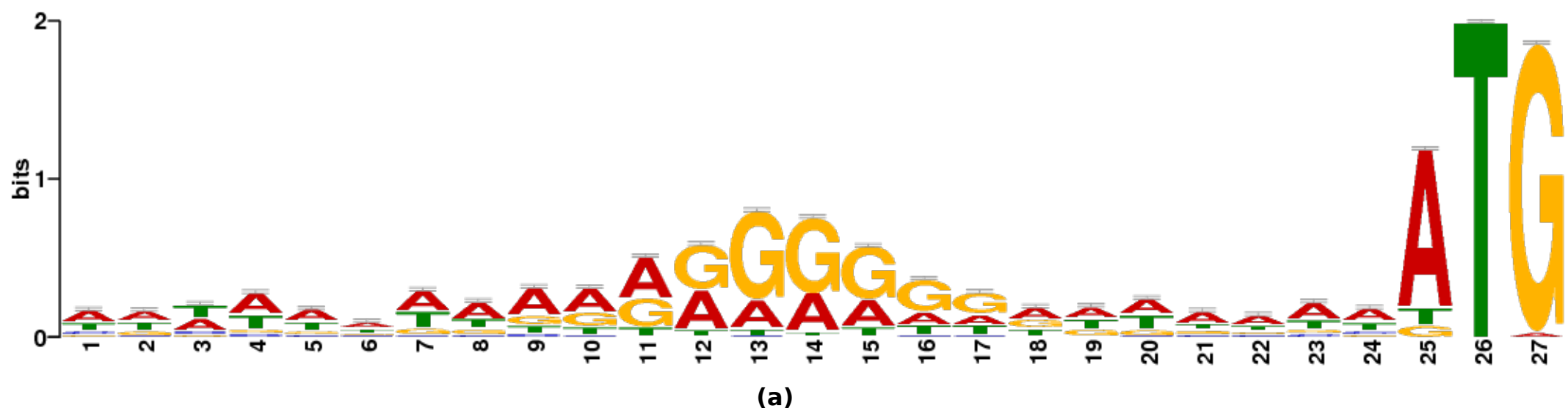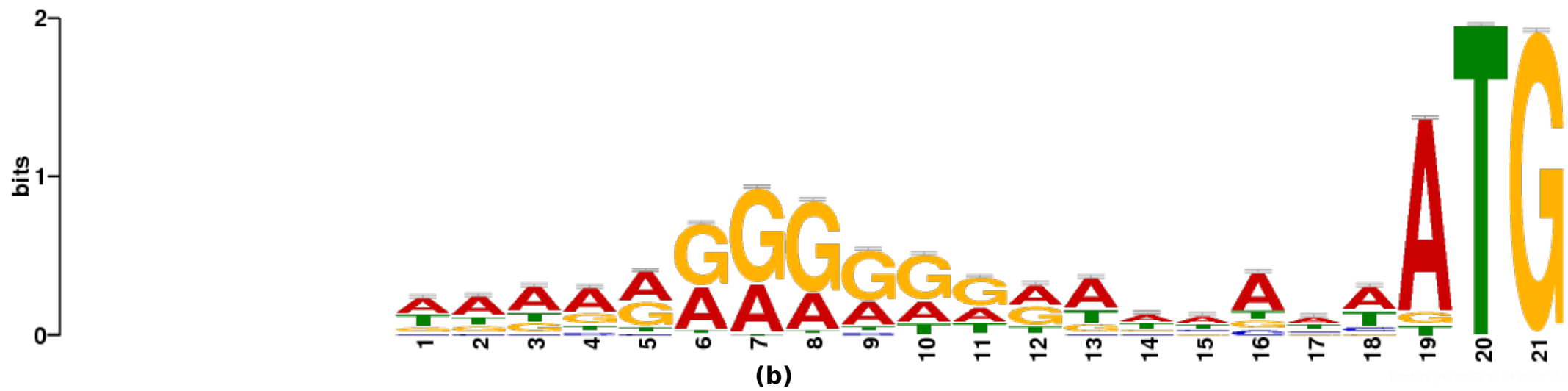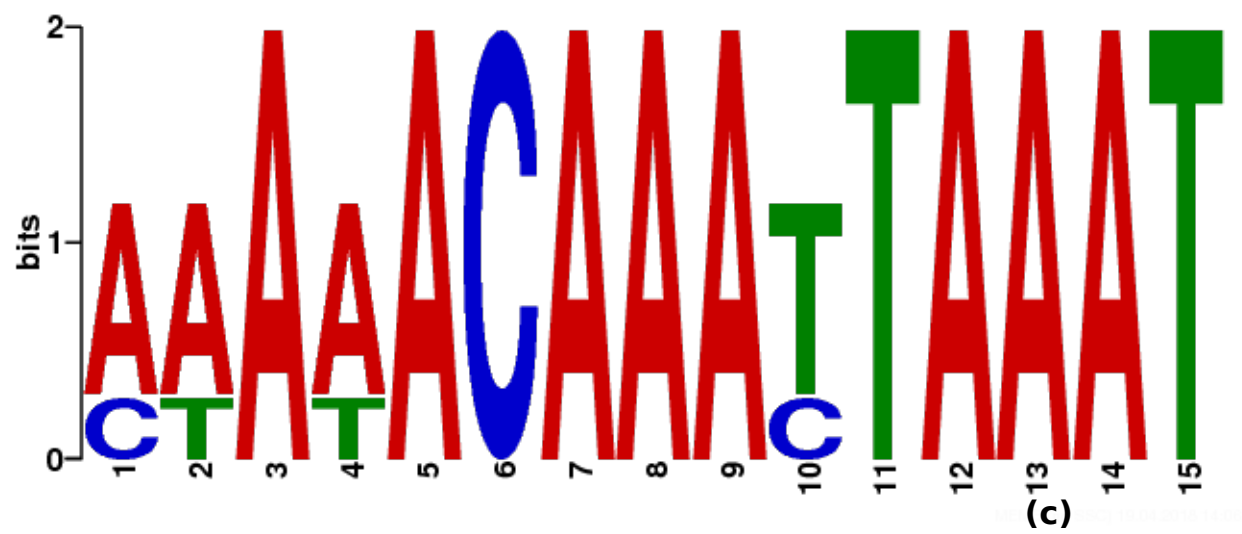

Supplement: Figure S1 — Motif logo for the identified promoters motif logo. (a) BM5 phage-RNP promoters motif logo, (b) BM10 phage-RNP promoters and Bacillus (host)-RNP promoters motif logo. [file peerj-06-5687-s007.pdf]

Cluster

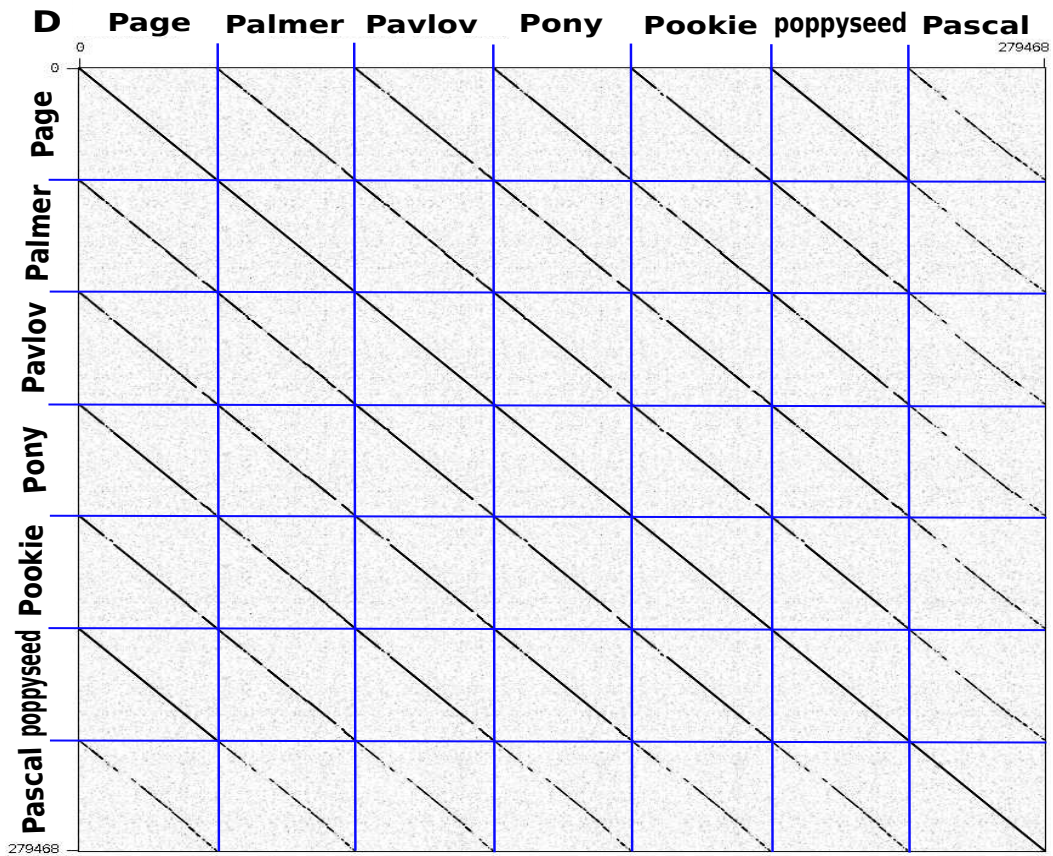

(A)

Cluster

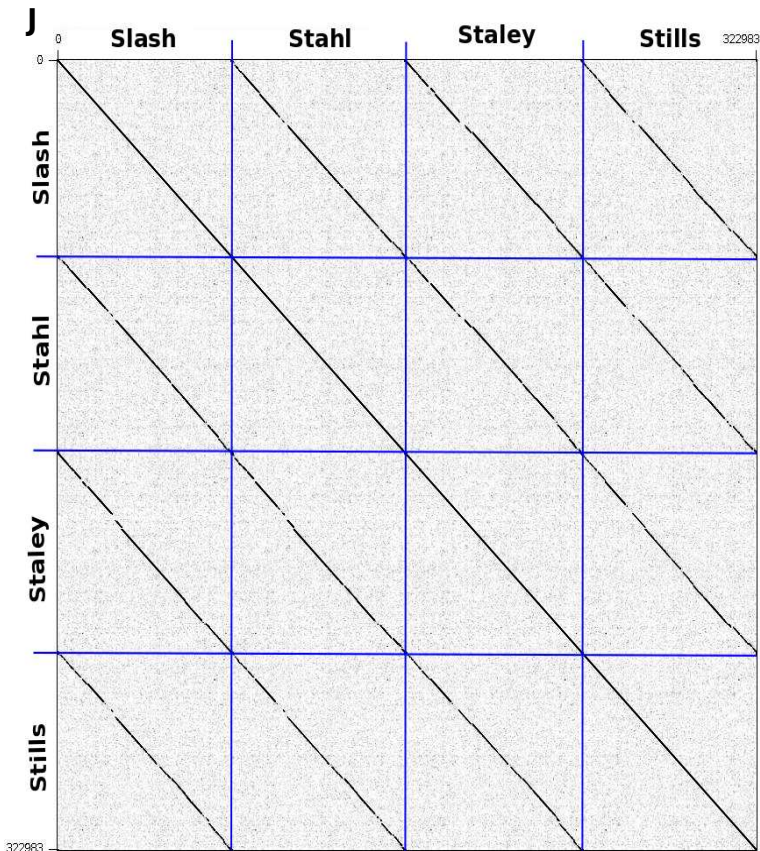

(B)

Cluster

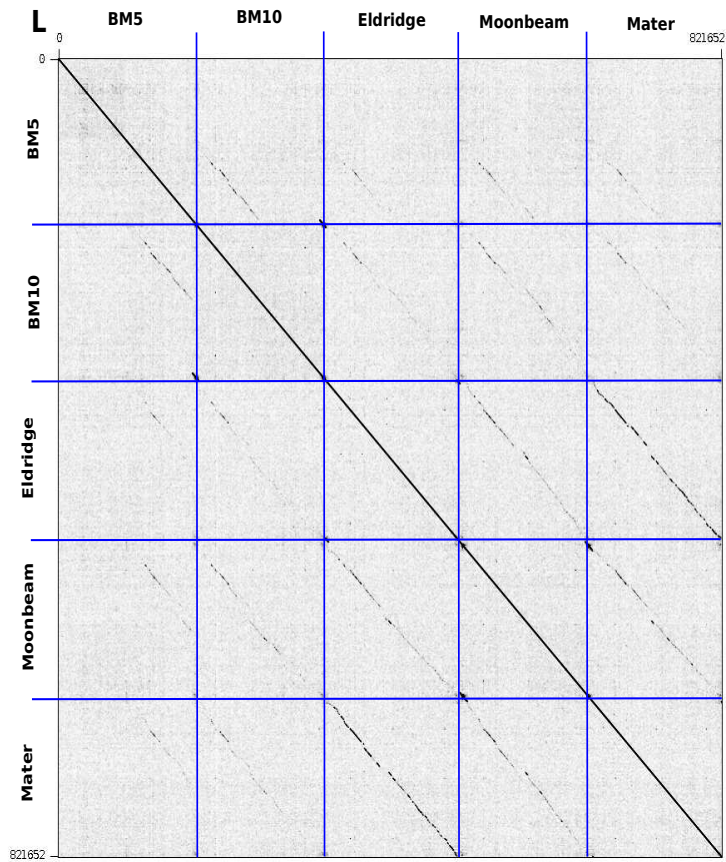

(C)

Supplement: Figure S2 — Analysis of fully sequenced Bacillus megaterium phage genomes belongs to clusters D, J and L. [file peerj-06-5687-s008.pdf]

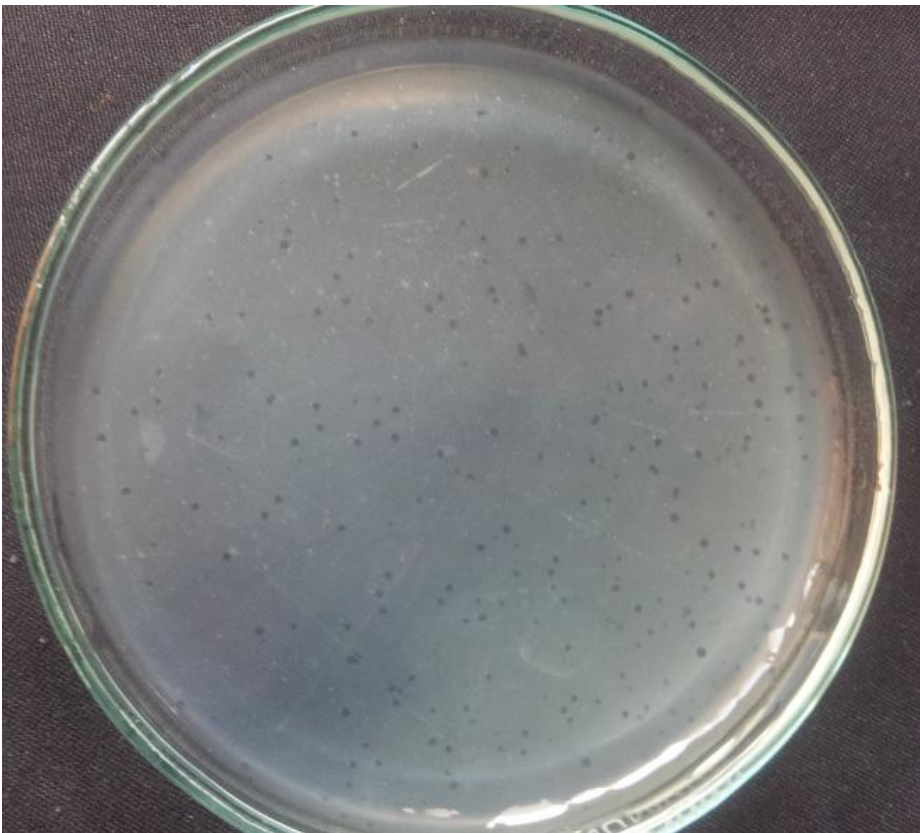

BM5

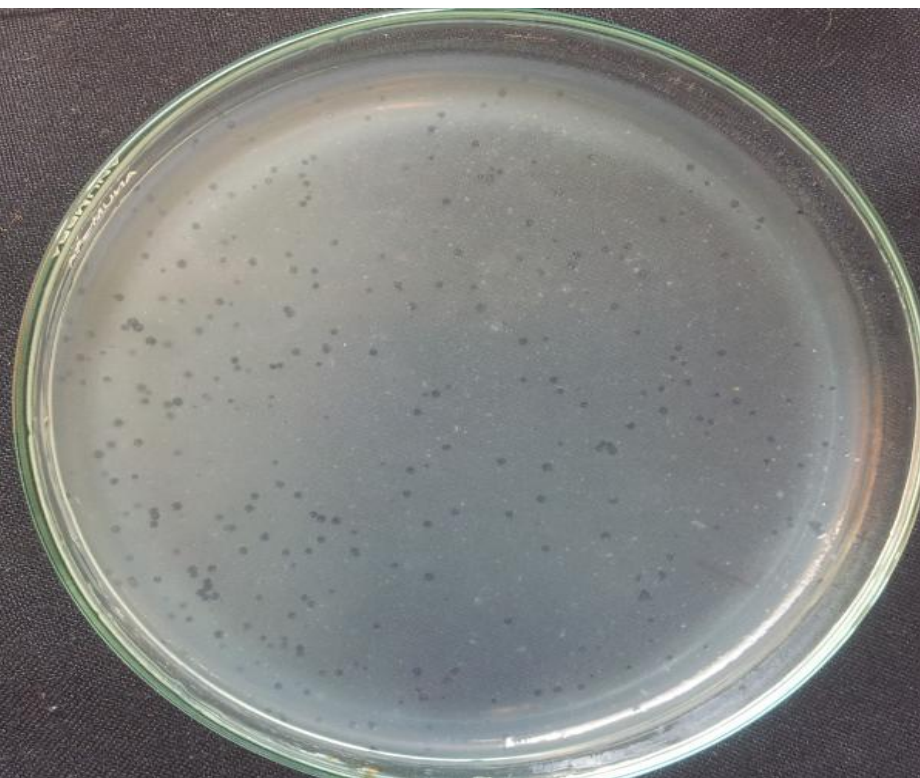

BM10

Supplement: Supplemental Information 2 — Original Plaques picture of the sequenced phages. [file peerj-06-5687-s010.pdf]
