# Supplementary material for: Characterization and comparative genomic analysis of virulent and temperate Bacillus megaterium bacteriophages
Source: PeerJ. 2018 Dec 10;6:e5687. doi: 10.7717/peerj.5687 (PMC6292376; doi:10.7717/peerj.5687)
Supplement: Supplemental Information 1 — Single-step experiment raw data. [file peerj-06-5687-s009.pdf]

Number of phagers per µl
Pfu/µl
Time (min.)

**Phage**	15	20	25	30	35	40	45	50	55	60	65	70	75
**BM5**	10	10	10	45	124	295	400	700	1030	1030	1030	1030	1030
**BM10**	19	19	19	19	55	190	420	620	910	1200	2223	2223	2223

Log. number of phagers per µl
Log. Pfu/µl
Time (min.)

**Phage**	15	20	25	30	35	40	45	50	55	60	65	70	75
**BM5**	4	4	4	4.7	5.1	5.4	5.6	5.8	6.01	6.01	6.01	6.01	6.01
**BM10**	4.3	4.3	4.3	4.3	4.7	5.2	5.6	5.7	5.9	6.07	6.4	6.4	6.4
